# Optimizing Global Navigation Satellite Systems network real-time kinematic infrastructure for homogeneous positioning performance from the perspective of tropospheric effects

**DOI:** 10.1098/rspa.2020.0248

**Published:** 2020-10-14

**Authors:** Chen Yu, Nigel T. Penna, Zhenhong Li

**Affiliations:** 1 School of Engineering, Newcastle University, Newcastle upon Tyne NE1 7RU, UK; 2 College of Geological Engineering and Geomatics, Chang'an University, Xi'an 710054, People's Republic of China; 3 Key Laboratory of Western China's Mineral Resource and Geological Engineering, Ministry of Education, Xi'an 710054, People's Republic of China

**Keywords:** Global Navigation Satellite Systems, network real-time kinematic, continuously operating GNSS reference stations, station spacing, water vapour, precise positioning

## Abstract

Real-time centimetre-level precise positioning from Global Navigation Satellite Systems (GNSS) is critical for activities including landslide, glacier and coastal erosion monitoring, flood modelling, precision agriculture, intelligent transport systems, autonomous vehicles and the Internet of Things. This may be achieved via the real-time kinematic (RTK) GNSS approach, which uses a single receiver and a network of continuously operating GNSS reference stations (CORS). However, existing CORS networks have often been established simply by attempting regular spacing or in clusters around cities, with little consideration of weather, climate and topography effects, which influence the GNSS tropospheric delay, a substantial GNSS positional error and which prevents homogeneous RTK accuracy attainment. Here, we develop a framework towards optimizing the design of CORS ground infrastructure, such that tropospheric delay errors reduce to 1.5 mm worth of precipitable water vapour (PWV) globally. We obtain average optimal station spacings of 52 km in local summer and 70 km in local winter, inversely related to the atmospheric PWV variation, with denser networks typically required in the tropics and in mountainous areas. We also consider local CORS network infrastructure case studies, showing how after network modification interpolated PWV errors can be reduced from around 2.7 to 1.4 mm.

## 1. Introduction

Real-time Global Navigation Satellite Systems (GNSS) positioning with centimetre-level precision and accuracy is commonly used and needed in utilities mapping [1], landslide monitoring [2], rapid earthquake source determination and tsunami early warning [3], coastal erosion monitoring [4], glacier and permafrost monitoring [5,6], river channel monitoring [7], precision agriculture [8,9], highway construction [10], structural monitoring [11], flood modelling [12], and photogrammetric mapping and LiDAR surveys from aeroplanes/helicopters [13] and unmanned aerial vehicles [14]. Most of these applications and studies have realized such centimetre-level positioning using local, single baseline real-time kinematic (RTK) GNSS. However, this has the limitation that the user must establish their own GNSS reference station within about 10 km of the user's roving receiver [15], so that common user and reference station GNSS errors such as atmospheric delay can be eliminated by data differencing.

In parallel with the development of new GNSS that complement and enhance the original Global Positioning System (GPS), notably GLONASS, Galileo and BeiDou, networks of continuously operating GNSS reference stations (CORS) have been increasingly established in many parts of the world. This has led to the generation of real-time precise global satellite orbits and clocks which may then be held fixed by a user's receiver to enable centimetre to decimetre-level positioning with a single receiver via the Precise Point Positioning (PPP) technique. While PPP does not require the direct use of CORS data, the drawback is that the unknown number of carrier phase cycles between the satellite and receiver (the integer ambiguity) is estimated as a floating point value, and it usually takes tens of minutes for such float ambiguities to converge to the correct values and thereafter centimetre to decimetre positional accuracy to be realized [16]. An enhancement to float PPP is PPP-RTK, whereby in addition to the fixed global satellite orbits and clocks, corrections from nearby CORS are transmitted and used to reduce atmospheric errors and to fix the float ambiguities to integer values to achieve improved positional accuracy and convergence times, but this can still take 10–20 min [17]. In the meantime, the traditional local RTK has been enhanced by the use of CORS, namely Network RTK, to achieve real-time single receiver positioning with centimetre-level accuracy. Network RTK does not require global precise satellite orbits and clocks or require the user to establish their own local GNSS reference station, but makes use of a regional network of CORS at known ground locations where observational error corrections, such as for orbits, ionospheric and tropospheric delays [18–22], are continuously computed and transmitted to the user's roving receiver in real time. A further Network RTK development is single-epoch (instantaneous) positioning as demonstrated by Paziewski [21], involving the resolution of the integer ambiguities and estimation of positions independently each epoch [23]. This single-epoch approach eliminates the effect of receiver cycle slips and has been used for the detection of, for example, teleseismic surface waves [24], and is now also being applied for multi-GNSS constellation positioning [25]. Therefore, Network RTK currently plays an important role in all the above-mentioned applications, and also in developing technologies which require real-time seamless, reliable, instantaneous positioning with centimetre-level precision and accuracy, such as intelligent transport systems [26], autonomous vehicles and the Internet of Things.

Current CORS networks (potentially) used for Network RTK positioning have been established in a relatively ad hoc manner. In some instances, such as in the British Isles, Sweden and Victoria (Australia), Network RTK service providers make use of governmental geodetic networks of CORS which have been set up with approximately even geographical spacing (e.g. 60–80 km in Britain). However, in many nations, such CORS are clustered in urban areas or along highways (e.g. Australia and Canada), or to provide national geodetic infrastructure for goals such as determining plate motions, earthquake characteristics and volcano dynamics, and hence many such CORS are concentrated around faults or plate boundaries (e.g. the Network of the Americas (NOTA) [27]: https://www.unavco.org/projects/major-projects/nota/nota.html). Many other Network RTK service providers have established their own specific commercial CORS networks, although these also tend to comprise either urban area clusters or an approximately even geographical spacing. Regardless of the set up, Network RTK works on the premise that the application of the transmitted, interpolated errors from the CORS enable the same centimetre-level user positional quality to be obtained as from local reference station RTK positioning. However, little apparent consideration has been given to whether the established CORS are optimally spaced with regard to the ensuing positional accuracy at the user's location, and there is a dearth of information on the optimal CORS configuration needed and whether dominant error sources, notably atmospheric delays, can be sufficiently interpolated and mitigated with existing CORS infrastructure.

Atmospheric delays arise from both the ionosphere and the neutral atmosphere (troposphere). The ionospheric delay used in Network RTK positioning may be estimated from dual- or multi-frequency phase and pseudorange observations at CORS, interpolated to the user's rover station and applied directly or as weighted pseudo-observations [21,28]. Ionospheric activity is greatest over the tropics and polar regions and depends on an 11-year sun spot cycle and space weather [29], while tropospheric activity is largely governed by both rapid spatial and temporal variations in atmospheric precipitable water vapour (PWV) [30], which is dependent on the Earth's climate, weather and topography. Tropospheric delays may be estimated at CORS using a random walk process [31] and, as with ionospheric delays, interpolated to the user's rover location and then applied directly. However, unlike ionospheric delays, tropospheric delays are topography-dependent and space and terrestrial weather are totally different. Therefore, the same interpolation approach cannot necessarily be used for both ionospheric and tropospheric delays, and hence the optimal CORS spacing can differ. In this paper, we investigate the optimal Network RTK CORS distribution for tropospheric delay determination. Our main objective is to determine how many CORS are required over a given region, by computing the station spacing density needed for the successful interpolation of PWV (and hence tropospheric delay) with homogeneous accuracy, and to investigate the dependence on geographical location, topography and climate. Ensuring such optimal CORS network design for tropospheric delay will help enable homogeneous performance of Network RTK and subsequently its practical uptake for all of the aforementioned RTK applications and developing technologies.

We consider the impact of PWV global spatial (climatic) and temporal (seasonal) distributions in formulating CORS network optimal design, as well as topographic variations. Scenarios for idealistic new CORS Network RTK design are considered, ranging from station spacings of 80 to 5 km (assumed to be the maximum density of a CORS network anywhere), along with case studies from selected national CORS networks illustrating how existing infrastructure can be best densified to mitigate tropospheric delay errors.

## 2. Precipitable water vapour reference dataset

To provide a means of evaluating the optimal CORS spacing for PWV interpolation, data from the Moderate Resolution Imaging Spectrometer (MODIS) instrument on board NASA's Terra and Aqua satellites were used to obtain a reference set of PWV values globally at approximately 5 km (0.05° grid) spatial resolution, in accordance with the assumed maximum CORS network density. This meant that various station spacing configurations could be evaluated by thinning the reference dataset and interpolating PWV with different station spacings and comparing with the reference PWV values. The near-infrared band water vapour MODIS product [32] which we used has a PWV accuracy and precision of about 1–2 mm in cloud-free conditions based on comparisons with GPS and radiosondes [33,34], so can be considered a proxy for GNSS-derived PWV. It provides higher accuracy than PWV from high-resolution global and regional numerical weather models [30,35], particularly over regions where few PWV observations have been assimilated, such as Africa.

**Figure 1.**
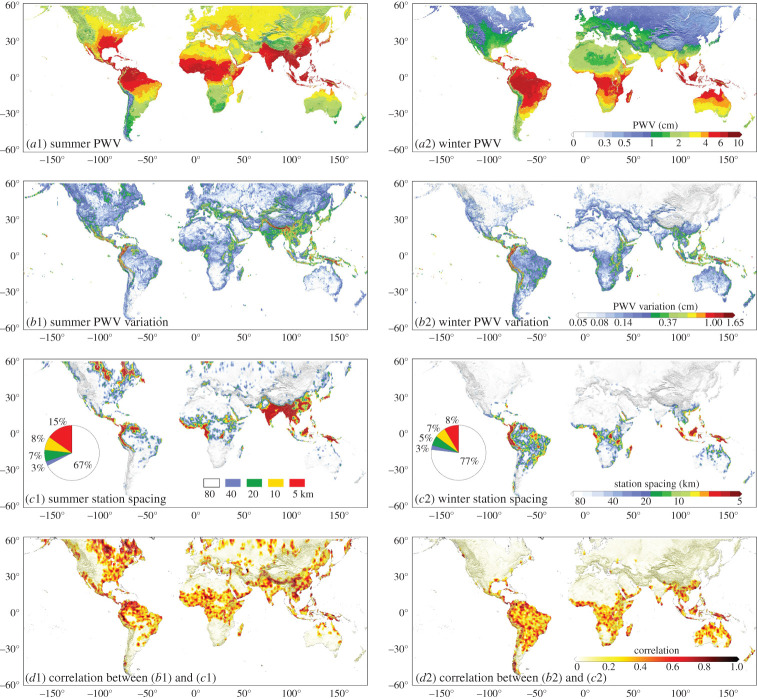
The simulated global GNSS network distribution optimization for boreal summer (June–August 2016) and boreal winter (December 2015–February 2016). (*a*) Average PWV content from MODIS (0.05° resolution). (*b*) PWV variations, defined as the PWV standard deviation of all points within a 0.8° × 0.8° window centred about each 0.05° MODIS pixel (0.05° resolution). (*c*) Optimal station spacing (0.8° resolution), including percentage of grid cells with different spacing. (*d*) Correlation map between the optimal station spacing and PWV variation (0.8° resolution). Topography variations are denoted by the shaded background. (Online version in colour.)

The MODIS product we used comprised PWV data at about 1 km spatial resolution, with 1–2 days required for satellite measurements of the whole Earth's surface. As the approximately 1 km data were not uniformly spaced, we sampled them to a regular 0.05° × 0.05° grid (approx. 5 km spacing), taking as the grid node value the nearest original (1 km) data point. To reduce the impact of meridional convergence on the uniformity of the grid point spacing, we only used in our tests grid points from the equator to latitude 60° north/south, at which 0.05° grid spacing corresponds to approximately 5.6 km and approximately 2.8 km, respectively. The resulting latitude 60° north to south PWV distributions over land are shown in figure 1 for three-month averages for boreal summer (1 June 2016–31 August 2016) and for boreal winter (1 December 2015–29 February 2016). This averaging per grid point was done to reduce the effect of weather variations during each season, but also to minimize the effect of cloud-induced data availability gaps. PWV measurements over water bodies were excluded using the Shuttle Radar Topography Mission 90 m digital elevation model (DEM) [36], and a DEM corresponding to the 0.05° PWV grid was generated to enable the assessment of topographic effects on PWV interpolation.

## 3. Interpolation of precipitable water vapour

The subsequent CORS network spacing tests were based on PWV interpolation using the iterative tropospheric decomposition (ITD) method. This was developed by Yu *et al*. [37], demonstrating a 1.7 mm PWV interpolation accuracy across both flat and mountainous terrain, and in both summer and winter. The ITD method has been used extensively for computing tropospheric corrections for Interferometric Synthetic Aperture Radar (InSAR) studies, including from GPS stations [38] and from numerical weather models [39], and used, for example, for improving measurements of volcano deformation [40] and co-seismic deformation [41]. The ITD method is based on the assumption that PWV comprises a stratified (topography/elevation-dependent) component, and a turbulent component, representing topography-independent PWV signals (below equation)

3.1 $$\text{PW}{\text{V}}_{i}=S(H_{i})+T({\text{x}}_{i})+\varepsilon_{i}$$

where $S(H_{i})=L_{0}{\text{e}}^{-\beta H_{i}}$ and represents the stratified component at position vector ${\text{x}}_{i}$; *i* is the location considered; $H_{i}$ is the height above sea level; *L*_0_ is the stratified PWV at sea level; *β* is an exponential coefficient; *T* is the turbulent PWV component; $\varepsilon_{i}$ is the residual PWV.

As described mathematically in [37,39], to interpolate the PWV to a specific point location, the ITD method takes as input all PWV values within a defined tropospheric decorrelation distance (here 150 km as per [39]) from the location considered. The PWV values are used to estimate an initial value for the stratified component, computing *L*_0_ and *β* and setting the turbulent component to zero. The residuals (differences between the PWV values per point and the initial value for the stratified component) are then computed, which are deemed to contain the turbulent component plus any unmodelled stratified PWV component. The residuals are then interpolated using inverse distance weighting to obtain an initial estimate of the turbulent component at the location of interest, and repeated to obtain initial values for the turbulent component for all PWV points, and subtracted from the initial values of the stratified delay per point. The stratified delay is then re-estimated to obtain updated values for the exponential coefficients and subsequently updated residuals, which are interpolated again to obtain updated values for the turbulent component. This iterative process is repeated until the estimates for the stratified and turbulent PWV components converge, and the final value of the interpolated PWV is obtained by summing the stratified and turbulent components, plus the residual. Typically, six or seven iterations are needed, as demonstrated in [37]. Importantly, from the analysis of a preliminary station spacing assessment over California, Yu *et al*. [38] found with the ITD model that a 5 km spacing (as used for the reference MODIS PWV dataset here) resulted in a similar accuracy as using a 2 km spacing for the interpolation of PWV, with interpolation errors from both spacings at the noise level of the PWV measurements.

## 4. Global optimal station spacing

We used the global three-month boreal summer and winter approximately 5 km MODIS PWV datasets to evaluate the optimal CORS network distribution globally across the land masses separately for both summer and winter, based on PWV interpolation using the ITD method. This was achieved by first sampling the PWV values from the MODIS 5 km global resolution reference dataset to 80 km (0.8°) resolution, indicative of the typical maximum spacing between Network RTK CORS in many nations. Then the CORS spacing needed in each cell of the 0.8° × 0.8° grid was determined by interpolating to 5 km resolution within each cell using a quadtree decomposition method [42] and cross-validating with the reference 5 km grid PWV values. If the RMS of the differences between the interpolated and reference PWV values per 5 km point within the 0.8° × 0.8° grid cell was greater than 1.5 mm, the station spacing within the particular cell was densified until the RMS reduced below 1.5 mm or the assumed densest 5 km station spacing was reached. A PWV cut-off error of 1.5 mm was chosen as it is equivalent to a zenith tropospheric delay error of about 1 cm [43], which maps to a GNSS height error of about 2–3 cm [44], the typical precision of RTK positioning [8,45,46].

The specific approach to determine the station spacing needed per 0.8° × 0.8° grid cell was to first subdivide each 0.8° × 0.8° grid cell into 256 (16 × 16) pixels which matched the 0.05° MODIS PWV reference grid. Then PWV values per pixel were obtained by interpolation from the PWV values of the 0.8° station spacing grid and which fell within the 150 km decorrelation distance of the pixel considered. The RMS of the differences between the interpolated and reference value for each of the 256 pixels per 0.8° × 0.8° cell was computed, resulting in a set of RMS PWV interpolation errors per cell of a 0.8° grid globally. If in any cell the RMS error was greater than 1.5 mm, the 0.8° station spacing used was considered insufficient for that particular cell and densified until the RMS error for each and every cell was less than 1.5 mm. This was realized by densifying such grid cells to use 0.4° reference PWVs for interpolation, or a station spacing of approximately 40 km. The interpolation to the 256 pixels in the 0.8° cell was then repeated, but using the 0.4° grid points within the 0.8° cell, plus the 0.8° grid points outside it. If the RMS error for the same 256 pixels was still greater than 1.5 mm, the 0.8° grid cell was further densified to a simulated station spacing of 0.2° (approx. 20 km), and the interpolation repeated. This was continued if necessary using 0.1° then 0.05° spacing until the RMS error for all 256 pixels was lower than 1.5 mm, or the 0.05° reference spacing was reached. The station spacing needed per 0.8° grid cell was then deemed the coarsest grid spacing needed for the RMS error to be 1.5 mm or lower. The resulting station spacing map shown in figure 1 consequently comprises a station spacing per 0.8° cell ranging from 80, 40, 20, 10 to 5 km.

Clear seasonal variations were obtained in the optimal station spacing, with a denser network being required in summer than in winter. The densest station coverage of 5–10 km is needed in the Andes, Central America, the Sierra Pacaraima, West Africa and the East Indies throughout the year. Other regions with very dense spacings of 5–10 km in summer are India and southeast Asia (Burma, Thailand, Vietnam, Cambodia, Laos, southern China and southern Japan), as well as parts of the Alps. Over much of Asia, Europe, North America, northern and southern Africa, southern South America and Australia, the sparsest station spacing of 80 km is sufficient. As listed in table 1, the average station spacing globally (defined as being the land masses from 60° north to 60° south) is 52 km in local summer and 70 km in local winter. The spacing, in general but not exclusively, tends to be denser in the tropics (average spacing of 46 km) than in cooler regions.

**Table 1.** Determined optimal station spacings for the global and regional network simulation.

	average station spacing (km)	median station spacing (km)	minimum station spacing (km)	maximum station spacing (km)
global network simulation (local summer^a^)	52	51	5	80
global network simulation (local winter^b^)	70	72	5	80
global network simulation (tropical areas^c^)	46	46	5	80
British Isles network^d^	48	46	5	71
Italy network	38	37	4	80
New Zealand network	35	33	1	45
Java network	6	6	5	22

^a^June–August 2016 for the Northern Hemisphere and December 2015–February 2016 for the Southern Hemisphere.

^b^December 2015–February 2016 for the Northern Hemisphere and June–August 2016 for the Southern Hemisphere.

^c^23.5° S to 23.5° N.

^d^Local summer of 2016: July 2016 for the British Isles and Italy, January 2016 for New Zealand and Java.

**Figure 2.**
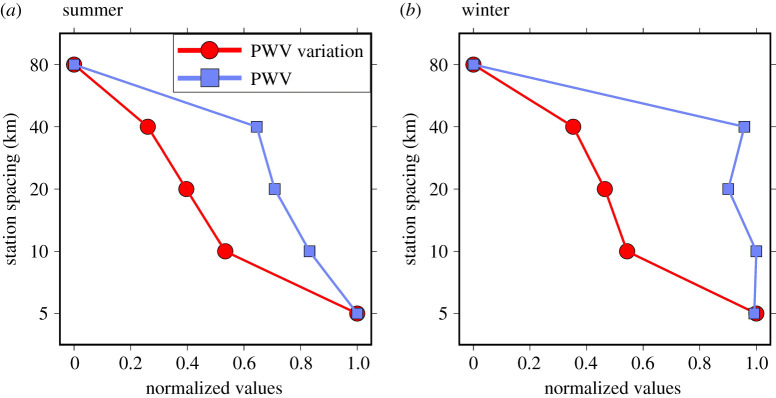
Relationship between the PWV and station spacing (blue line), and between the PWV variation and station spacing (red line), for local summer (*a*) and local winter (*b*), respectively. The PWV or PWV variation is a global average of all pixels at the corresponding spacing, and is normalized linearly between (0, 1). (Online version in colour.)

To understand the cause of the variation in the optimal station spacing, included in figure 1 are maps of three-monthly average PWV per 0.05° MODIS pixel. It can be seen that many of the areas with the densest station spacing occur where the PWV content is very high (approaching 10 cm), notably in the tropics and across India and southeast Asia in local summer. However, there are also regions which have a high PWV content, such as central Africa and the Amazon basin, yet the station spacing needed to accurately interpolate PWV is very sparse. By comparing the maps of the station spacing with maps of PWV variation (also shown in figure 1), defined as the standard deviation of all 256 0.05° grid PWV values within an 80 × 80 km (0.8° × 0.8°) window centred on each 0.05° MODIS pixel considered, it can be seen that it is the PWV spatial variation that has more influence on the station spacing than the PWV magnitude itself. We also computed correlation coefficients between station spacing and PWV variation for each 0.8° grid cell, using the station spacing and PWV variation for the cell itself and the eight 0.8° cells immediately surrounding it. The plot of the resulting correlations shown in figure 1 confirms the influence of PWV variation on station spacing. For example, high correlation values between station spacing and PWV variation are seen around the Andes, west Africa and southeast Asia, and the Alps in local summer. Meanwhile, for the Amazon basin, there is little correlation because the PWV variation across the region is small, and the station spacing is sparse. Furthermore, in figure 2, plots of PWV and PWV variation (normalized linearly to (0, 1) against the respective global minimum and maximum) against station spacing are shown, collated for all global values for both local summer and winter. It can be seen that there is a clear inverse linear relationship between the normalized PWV variation and station spacing for both summer and winter, but the normalized PWV itself has weak correlations (if there is any) with station spacing, especially in winter.

One of the principal causes of large spatial variations in PWV above the ground at a particular location, and hence the dense station spacing, is the topography. The Andes, located at a similar latitude to the Amazon basin, exhibit rapid and large changes in topography over distances of only a few kilometres, and, therefore, rapid PWV variations because PWV content scales with height. Similar effects are seen over the Alps in local summer (but not in adjacent flatter regions such as France and Germany), and in particular in Nepal and Bhutan where the Himalayas meet the Indian plain. The terrain is flatter across much of India, but the monsoon climate means extreme variations in PWV can rapidly occur, and hence the need for a dense station spacing. However, in local winter in the same parts of India, only the minimal sparse spacing of 80 km is needed, as the PWV variations are only moderate (0.14 cm). Although figure 1 indicates that a dense station spacing is needed in northeast Canada in local summer, the large PWV variations there are not considered reliable because the MODIS PWV values are degraded by the large number of lakes, whose average size is several kilometres and close to the MODIS spatial resolution [32]. In local winter however, the average PWV content is low (approx. 0.4 cm), as is its variation (approx. 0.08 cm), meaning that the effect of the lakes is small and the sparsest 80 km spacing is always sufficient.

## 5. Local continuously operating GNSS reference stations network infrastructure optimization and enhancement

The optimal station spacing globally has been considered by assuming a regular station grid and without considering existing geodetic GNSS infrastructure. In practice, there are a large number of CORS stations already existing and, while in some cases, they have been designed to form as regular a station spacing as possible (e.g. about 60–80 km in Britain), invariably they are irregularly spaced, and designers do not seem to have considered topographic or climatic variations. Consequently, existing networks do not necessarily provide optimal PWV mitigation performance. Here, we provide a method of how to densify such existing CORS infrastructure to obtain optimal PWV interpolation performance with the fewest additional new stations and without having to redesign the network completely (hence new CORS are added to the existing networks, not replacing them). We then illustrate the concept with some examples from different parts of the world, which are simply indicative based on existing CORS infrastructure as of December 2016: only in some cases, do they currently underpin commercial and/or free Network RTK services. Nevertheless, they provide indications of how infrastructure may be densified if all stations were able to be used for Network RTK services.

To assess whether existing GNSS networks are optimally spaced, or what CORS densification should be carried out, a similar interpolation approach to the global uniform grid simulations was used. However, as existing GNSS networks have an irregular distribution of stations, instead of starting from a coarse uniform grid, we formed a Triangulated Irregular Network (TIN) based on Delaunay triangulation [47]. The other difference was that one-month averages of the MODIS PWV daily values per point of the global 0.05° grid were used, not three-month averages. The procedure comprised:

(i) A TIN was constructed using the original network, i.e. using all existing stations, where the PWV value for the station was taken as that of the nearest 0.05° grid point.
(ii) The PWV from the nodes of the TIN were interpolated on to the 0.05° grid reference points using the ITD model.
(iii) The RMS error (difference between the interpolated and reference PWV values) of all the 0.05° grid points contained within each triangle of the TIN was computed and, where the RMS error exceeded 1.5 mm, a new station at its geometric centre was inserted.
(iv) The TIN was reconstructed based on the original and added stations, and steps (i) to (iii) repeated until the RMS error of the 0.05° grid points within each and every triangle was not greater than 1.5 mm.

**Figure 3.**
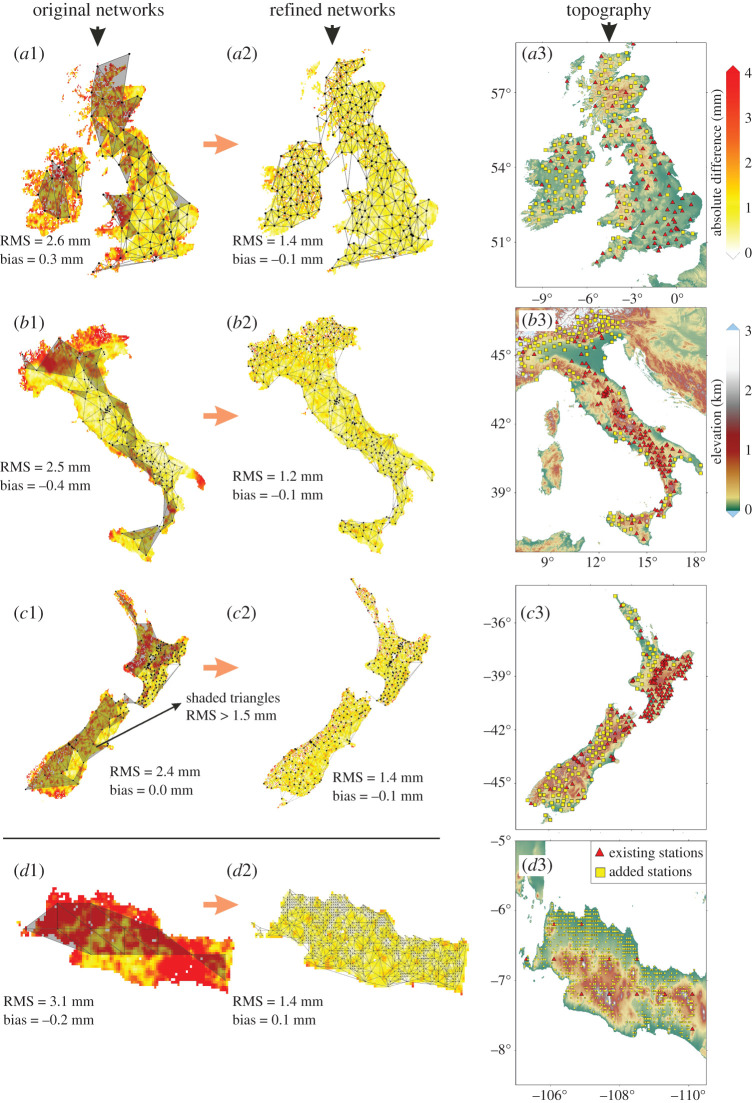
Original and densified (optimally refined) regional networks during local summer of 2016: July 2016 for the British Isles and Italy, January 2016 for New Zealand and Java. The first (*a*1–*d*1) and second (*a*2–*d*2) columns represent the original and the densified networks, respectively, with the shaded triangles indicating areas where the interpolation RMS exceeds 1.5 mm. The coloured background underneath the TIN of the first two columns shows the (absolute) differences between the PWV values based on interpolation from the reference stations and the reference MODIS values. The third column (*a*3–*d*3) shows the topography and the additional stations inserted to obtain the optimal spacing. (Online version in colour.)

To illustrate the network densification concept, four examples from existing networks are considered. The first example is the British Isles (www.bigf.ac.uk), as the bulk of the network comprises national CORS geodetic infrastructure used for Network RTK service provision and has a near regular station spacing of about 60–80 km (figure 3). It exhibits moderate PWV variations and fairly flat topography in England, but with more mountainous terrain in Scotland and Wales, albeit only rising to a maximum of about 1300 m elevation. Note that some evaluations of the positional accuracy from Network RTK commercial services using CORS from this British Isles network are given in [48]. Accuracies of 1–2 cm in plan and 1.5–3.5 cm in height were obtained from 6 h of continuous data, with accuracy tending to be poorest at greater elevation differences from nearby CORS. The second example considers Italy's RING network (http://ring.gm.ingv.it/), which has stations clustered as densely as every 25 km in the Apennines, but in the north, the distribution is much sparser at around 80 km, especially in and close to the Alps where particularly large PWV variations occur in local summer. The third example is New Zealand's GeoNet (https://www.geonet.org.nz), which incorporates dense station spacing (approx. 25 km) in the east of the north island. However, spacing is much sparser (approx. 90 km) across the Southern Alps in the south island, which exhibit a large range in topography from about 3500 m elevation mountains to plains close to sea level. The fourth example is for western Java, which has very large PWV variations, large topographic variations from sea level to approximately 2000 m elevation and, from figure 1, very dense suggested station spacings of towards 5 km being needed. It is also chosen as an area with few existing CORS and as we are not aware of Network RTK services operating there, instead of showing how an existing network could best be densified, an initial hypothetical network with a regular 80 km spacing was assumed, and then densified.

The original (existing) and densified networks are shown in figure 3, considered for local summer (January or July 2016) for each network, when PWV variations are greatest. For the British Isles, 144 stations are added to the original 108. Across almost all of England, however, very few additional stations are needed with the original 60–80 km spacing almost sufficing. The additional stations are mainly in the mountainous areas of Wales and western Scotland, and also in Ireland where the assumed network is less dense originally. The new stations reduce the average inter-station spacing from 65 to 48 km (as listed in table 1), with an overall PWV RMS error reduction of 2.6–1.4 mm. In Italy, a very dense network of about 34 km spacing is needed in the Alps compared with the existing 80 km spacing, which has the effect of reducing the overall RMS error from 2.5 to 1.2 mm. Few additional stations are needed across the Appennines, as the PWV variations induced by the topographic variations are already well mitigated by the dense original network. Similar to Italy, for New Zealand, many (108) new stations (now with average spacing 40 km) are needed in the south island to obtain the optimal RMS error, while in the north island, those added tend to be in the flatter areas of the west coast but where there was a dearth of existing stations. The average spacing for all of New Zealand becomes 35 km, which is similar to the average of 38 km across Italy. For Java, a very dense network of 5 km station spacing is needed in many areas (with the average spacing being 6 km), but it can be seen from figure 3 that there are local variations in the spacing needed, including across parts of the mountains and adjacent flatter areas where PWV spatial variations are lower. Note that the minimum station spacing may be denser than our 5 km reference grid due to closely located stations in the original network, as arises for the Italy and New Zealand cases.

**Figure 4.**
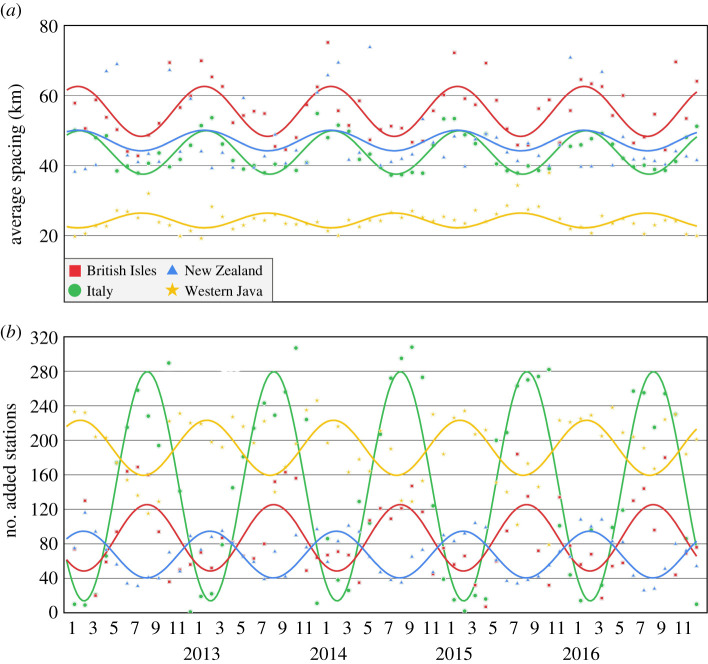
Time series of optimal station spacing from January 2012 to December 2016 for each of the British Isles, Italy, New Zealand and Java CORS networks. Shown are (*a*) the resultant monthly average station spacing and (*b*) the number of stations added to the original network to be optimal. Dots denote the actual station numbers per month per network. Solid lines show the best-fitting annual sinusoid per time series. (Online version in colour.)

A seasonal variation in the global optimal station spacing is suggested from the summer and winter PWV variation and station spacing maps shown in figure 1. To investigate the extent of such seasonal variations in optimal station spacing for the local network examples considered, the Delaunay triangulation optimal densification of the assumed existing networks was repeated for each month for 5 years from 2012 through to 2016. The number of additional stations needing to be inserted per network per month is shown in figure 4, together with the resultant average station spacing across the network. Clear periodic, seasonal variations in the number of added stations and resultant average station spacing needed for optimal PWV interpolation are seen. The amplitude of the seasonal variation is dependent on the type of climate, with the Mediterranean and Alpine climates of Italy exhibiting an amplitude of about 140 stations, while the tropical climate of Java has an amplitude of only 30 stations. The amplitude also provides an indication of how close the original network is to being optimal for Network RTK provision, as a small amplitude denotes that few stations need to be added, although it could also indicate that in local winter, the original network is denser than required in places and could potentially be thinned.

## 6. Conclusion and outlook

We have proposed and developed a framework to optimize GNSS CORS network infrastructure with regard to the distribution of the variations in PWV and topography, in order to facilitate centimetre-level homogeneous accuracy for Network RTK positioning. The optimal station spacings were found globally using MODIS PWV values as reference, and cross-validating after interpolation from station spacings ranging from 80 to 5 km. We have also presented more detailed local examples, demonstrating how assumed original existing CORS networks could be densified through a Delaunay triangulation approach in order to optimize the interpolation of PWV errors, with a clear seasonal variation demonstrated in the optimal network.

We have shown that the optimal station spacing of a CORS network for tropospheric delay mitigation depends on variations in PWV across the region considered, rather than the total amount of PWV. However, the largest variations, and hence the densest station spacings, tend to be in the tropical regions which have large PWV content, although topographic effects also have a major influence. For example, where large variations in topography arise such as in the Alps, a dense station spacing is needed. Local extreme weather also contributes, such as the monsoon climate of India which requires a very dense network in local summer despite being fairly flat, but only a fairly sparse network is needed in local winter. Summary statistics are provided in table 1.

The framework developed has been presented for the optimization of Network RTK GNSS positioning tropospheric delay errors. However, the resulting optimal CORS network designs are also important for InSAR data processing and applications, providing indications of whether existing GNSS stations will be able to provide sufficient quality corrections to reduce tropospheric effects on InSAR observations and hence improve estimates of post- and inter-seismic crustal movements [38]. Furthermore, the ability to generate precise maps of PWV from GNSS CORS data is also useful in meteorology in the nowcasting of rainfall [49] and assimilation into numerical weather models for precipitation forecasting [50,51]. Equally important, the framework concept developed here has the potential to be employed to optimize other monitoring networks, e.g. those for soil moisture, air pollution and tree species monitoring, although their own dominating error source should be identified and the corresponding spatial and temporal variations should be considered. In addition, there might be multiple dominating error sources in some cases, whereby the framework concept should be further developed to deal with multiple variables, such as both ionospheric and tropospheric delays when applied to Network RTK CORS infrastructure design.

## Data accessibility

The MODIS data used in this paper were gratefully obtained from the Land Processes Distributed Active Archive Centre (LP DAAC), managed by the NASA Earth Science Data and Information System (ESDIS) project (http://dx.doi.org/10.5067/MODIS/MOD05_L2.006).
